# Evaluación de tecnologías sanitarias para la toma de decisiones en Latinoamérica: principios de buenas prácticas

**DOI:** 10.26633/RPSP.2017.138

**Published:** 2017-11-07

**Authors:** Andrés Pichon-Riviere, Natalie C Soto, Federico Ariel Augustovski, Sebastián García Martí, Laura Sampietro-Colom

**Affiliations:** 1 Instituto de Efectividad Clínica y Sanitaria Instituto de Efectividad Clínica y Sanitaria Buenos Aires Argentina Instituto de Efectividad Clínica y Sanitaria, Buenos Aires, Argentina.; 2 Hospital Clinic de Barcelona Hospital Clinic de Barcelona Barcelona España Hospital Clinic de Barcelona, Barcelona, España.

**Keywords:** Cobertura universal, economía de la salud, salud pública, evaluación de la tecnología biomédica, políticas públicas de salud, asignación de recursos para la atención de salud, economía en atención de salud y organizaciones, prioridades en Salud, Universal coverage, health economics, public health, technology assessment, biomedical, public health policy, health care rationing, health care economics and organizations, health priorities, Cobertura universal, economia da saúde, saúde pública, avaliação da tecnologia biomédica, políticas públicas de saúde, alocação de recursos para a atenção à saúde, economia e organizações de saúde, prioridades em saúde

## Abstract

**Objetivo.:**

*Identificar los principios de buenas prácticas en la Evaluación de las Tecnologías Sanitarias (ETESA) más relevantes, aplicables y prioritarios en Latinoamérica; y las potenciales barreras para implementarlos en la región*.

**Métodos.:**

*Se identificaron los principios de buenas prácticas en ETESA postulados a nivel mundial y luego se exploraron mediante un proceso deliberativo en un Foro de evaluadores, financiadores y productores de tecnologías*.

**Resultados.:**

*El Foro contó con la participación de 42 representantes de diez países Latinoamericanos. Los principios de buenas prácticas postulados a nivel internacional fueron considerados válidos y potencialmente aplicables en Latinoamérica. Cinco principios fueron identificados como prioritarios y con mayor potencial para ser profundizados en estos momentos: transparencia en los procesos de realización de ETESA; Involucramiento de actores relevantes en el proceso de ETESA; existencia de mecanismos de apelación de las decisiones; existencia de mecanismos claros para el establecimiento de prioridades en ETESA; y existencia de un vínculo claro entre la evaluación y la toma de decisión. El principal reto identificado fue encontrar un equilibrio entre la aplicación de estos principios y los recursos disponibles para prevenir que las mejoras a introducir atenten contra los tiempos de producción de informes y la adecuación a las necesidades de los decisores*.

**Conclusiones.:**

*La principal recomendación fue avanzar gradualmente en mejorar la ETESA y su vínculo con la toma de decisión desarrollando procesos apropiados para cada país, sin pretender imponer a corto plazo estándares tomados de ejemplos a nivel internacional sin la adecuada adaptación al contexto local*.

## Abstract (en)

***Objective*.:**

Identify the most relevant, applicable, and priority good practice principles in health technology assessment (HTA) in Latin America, and potential barriers to implementing them in the region.

**Methods.:**

HTA good practice principles postulated worldwide were identified and then explored through a deliberative process in a forum of evaluators, funders, and technology producers.

**Results.:**

Forty-two representatives from ten Latin American countries participated in the forum. The good practice principles postulated at the international level were considered valid and potentially applicable in Latin America. Five principles were identified as priorities and as having greater potential to be expanded at this time: transparency in carrying out HTA; involvement of stakeholders in the HTA process; existence of mechanisms to appeal decisions; existence of clear mechanisms for HTA priority-setting; and existence of a clear link between assessment and decision-making. The main challenge identified was to find a balance between application of these principles and available resources, to prevent the planned improvements from jeopardizing report production times and failing to meet decision-makers’ needs.

**Conclusions.:**

The main recommendation was to gradually advance in improving HTA and its link to decision-making by developing appropriate processes for each country, without attempting to impose, in the short term, standards taken from examples at the international level without adequate adaptation to the local context.

## RESUMO

**Objetivo.:**

Identificar os princípios das boas práticas na avaliação de tecnologias em saúde (ATS) mais relevantes, aplicáveis e prioritárias na América Latina e as potenciais barreiras para implementação destes princípios na Região.

**Métodos.:**

Foram identificados os princípios das boas práticas na ATS propostos ao nível mundial e explorados em um processo deliberativo em um fórum de examinadores, financiadores e produtores de tecnologia.

**Resultados.:**

O fórum teve a participação de 42 representantes de 10 países latino-americanos. Considerou-se que os princípios das boas práticas propostos ao nível internacional são válidos e potencialmente aplicáveis na América Latina. Identificaram-se cinco princípios prioritários com maior potencial para serem aprofundados: transparência nos processos de ATS; envolvimento de atores relevantes no processo de ATS; disponibilidade de mecanismos de apelação das decisões; mecanismos distintos para determinação das prioridades em ATS; e vínculo evidente entre avaliação e tomada de decisão. Verificou-se que o principal desafio é encontrar um equilíbrio entre a aplicação dos princípios e os recursos disponíveis a fim de evitar que as melhorias a serem introduzidas sejam um obstáculo ao tempo de produção de relatórios e à adequação às necessidades dos responsáveis pela tomada de decisão.

**Conclusões.:**

A principal recomendação é avançar gradualmente para aperfeiçoar a ATS e o vínculo com a tomada de decisão, desenvolvendo processos adaptados a cada país, sem pretender impor a curto prazo padrões tidos como exemplares ao nível internacional sem a correta adaptação ao contexto local.

La cobertura universal en salud (CUS) es una prioridad a nivel mundial y una gran cantidad de países se han comprometido a alcanzarla para su población. Dados los recursos limitados de todo sistema de salud, esto implica tener que priorizar, y tomar decisiones sobre qué servicios serán provistos, para quienes, y a qué costo (1). En el año 2012 la Organización Panamericana de la Salud (OPS) y luego en el 2014 todos los estados miembros de la Organización Mundial de la Salud (OMS) aprobaron una resolución sobre Evaluación de Tecnologías Sanitarias (ETESA) y cobertura universal en salud en la cual se insta a fortalecer las capacidades de ETESA en los países, así como a integrar los conceptos y principios de la ETESA en las estrategias y áreas de trabajo en el camino hacia la cobertura universal en salud (2, 3). La ETESA es definida como un campo multidisciplinario de análisis de políticas, que evalúa las implicancias médicas, sociales, éticas y económicas del desarrollo, difusión y uso de una tecnología sanitaria (4).

El desarrollo de la ETESA ha sido notable en las últimas tres décadas a nivel mundial, y especialmente en los últimos 15 años en Latinoamérica y el Caribe (LAC); constituyendo hoy una parte indispensable de los sistemas de salud de varios países de la región (5). LAC cuenta ya con 29 instituciones de 14 países que integran RedETSA, la red regional de ETESA coordinada por la OPS, y son cada vez más los países de la región que utilizan reportes de ETESA para informar decisiones sobre asignación de recursos. Estos reportes de ETESA son realizados por una variedad de organizaciones, que incluyen agencias de ETESA, instituciones académicas, agencias regulatorias y los mismos productores de medicamentos y dispositivos.

El hecho de que la ETESA sea realizada y utilizada por una amplia variedad de actores, y que sea cada vez más vinculante en el proceso de toma de decisiones, ha hecho surgir una inquietud razonable por garantizar que tanto la forma en que se realiza la ETESA como la forma en que se aplica en el proceso de toma de decisiones sean las apropiadas para cada contexto. Si la ETESA no es realizada y utilizada de forma adecuada se corre el riesgo de producir tanto una asignación ineficiente de recursos, dando cobertura a intervenciones de poco o nulo beneficio, como de impedir o demorar el acceso de pacientes a tecnologías sanitarias útiles, y enviar mensajes equivocados a los productores de tecnologías (4). Por estos motivos, diversos grupos alrededor del mundo han identificado ejemplos de buenas prácticas y establecido recomendaciones y principios rectores en la ETE- SA (6–14).

La publicación de estos diferentes principios y recomendaciones ha promovido un importante debate, surgiendo las siguientes interrogantes (15–20): 1) ¿Son todos los principios de buena práctica igualmente importantes?; 2) ¿Es realista, o razonable esperar que todas las agencias de ETESA del mundo los satisfagan?; 3) ¿Todos deberían ser implementados de la misma forma por todos los sistemas de salud, o hay aspectos regionales o locales que hacen que muchos principios no sean aplicables para algunos contextos?

A esta controversia se suma que, en la mayor parte de los casos, estos principios de buenas prácticas fueron postulados por grupos provenientes de países de altos ingresos y pensados para ser aplicados principalmente en países con procesos y sistemas de ETESA más establecidos. Por otro lado, es habitual que estas recomendaciones provengan de grupos académicos, y no incorporen formalmente la visión de otros actores relevantes, como funcionarios de gobierno o financiadores, entre otros.

Este manuscrito presenta los resultados del Primer Foro Latinoamericano de Políticas de Evaluación de Tecnologías Sanitarias organizado por la Sociedad Internacional de ETESA (HTAi por sus siglas en inglés) (21). El objetivo de este foro fue identificar cuáles de los principios de buenas prácticas en ETESA que han sido postulados a nivel global podrían ser más relevantes, aplicables y prioritarios en LAC; qué barreras podría enfrentar la región para impulsarlos; y qué recomendaciones pueden hacerse a los sistemas de salud para mejorar la ETESA y su aplicación a la toma de decisiones en la región.

## MÉTODOS

El Foro de Políticas de Evaluación de Tecnologías Sanitarias es un método utilizado por HTAi para involucrar actores y emitir recomendaciones sobre temas concretos. Se realiza desde hace 13 años en Canadá, Estados Unidos y Europa, y en Asia desde hace cuatro años (22). El Foro se creó con el objetivo de proporcionar un espacio neutral para llevar a cabo discusiones de carácter estratégico sobre el estado de la ETESA, su desarrollo, y sus implicancias para el sistema de salud, la industria, los pacientes y otras partes interesadas. El Foro reúne a representantes de tres grupos principales de actores institucionales: 1) a quienes toman las decisiones sobre la cobertura y reembolso/precios de medicamentos y dispositivos en los sistemas de salud; 2) a los que realizan las ETESA en apoyo de esas decisiones; y 3) a las empresas biomédicas productoras de las tecnologías. En cada Foro se discute un tema de interés priorizado por todos sus participantes. Las discusiones del Foro se encuentran protegidas por las *Reglas del Chatham House* (23), las cuales permiten compartir fuera del Foro el contenido de los comentarios y discusiones llevadas a cabo, pero sin mencionar quiénes fueron sus autores. El cumplimiento de esta regla ha garantizado un clima de confianza y apertura entre los miembros que asisten año tras año al evento.

Para este Primer Foro de Políticas de ETESA de LAC, el Instituto de Efectividad Clínica y Sanitaria de Argentina (IECS – www.iecs.org.ar) actuó como la secretaría científica. La identificación del tema a discutir la realizó el Comité Organizador compuesto por representantes de las instituciones participantes (públicas y privadas) tras consultar a los miembros del Foro su preferencia sobre una lista de temas.

El tema seleccionado para este primer foro fue “Buenas prácticas en la aplicación de la Evaluación de Tecnologías Sanitarias en la toma de decisiones en Latinoamérica”.

La secretaría científica elaboró un “documento de base” resumiendo el estado de conocimiento sobre el tema para nutrir las discusiones. Para su confección se realizó una búsqueda dirigida a identificar documentos con información sobre principios y buenas prácticas en ETESA en términos generales. La estrategia incluyó bases de datos específicas (MEDLINE, CRD, LILACS), buscadores genéricos de internet, sitios web de instituciones relevantes y consulta con expertos. Se seleccionaron las publicaciones que presentaran criterios para la realización de ETESA interpretables como principios o buenas prácticas o que hicieran referencia a su aplicación en el mundo en general o en LAC en particular. Se excluyeron reportes y documentos dirigidos exclusivamente a cuestiones metodológicas. El documento base fue revisado por los participantes antes del evento y sus comentarios fueron valorados e incluidos.

El encuentro presencial del Foro se llevó a cabo en la ciudad de San José de Costa Rica los días 18 y 19 de abril de 2016. Una conferencia magistral a cargo de Sir Andrew Dillon, director ejecutivo del Instituto Nacional de Salud y Cuidados de Excelencia del Reino Unido (NICE, por sus siglas en inglés), y una serie de presentaciones sobre el estado de situación en la región a cargo de los participantes (agencias de ETESA, industria farmacéutica y de dispositivos, decisores y financiadores), sirvieron de marco para iniciar las discusiones. Los participantes se dividieron en cuatro grupos de discusión, con una distribución balanceada de los diferentes actores presentes en el evento. El primer día de actividades cada grupo trabajó sobre los principios de buenas prácticas postulados a nivel internacional con el objetivo de identificar aquellos en los que resultaría más prioritario enfocar los esfuerzos en LAC en los próximos años con base a dos criterios: 1) la relevancia del principio para mejorar los procesos de ETESA en la región y 2) la existencia de mayores brechas entre la situación ideal y el grado actual de implementación de dicho principio. Adicionalmente, de los principios priorizados, los participantes identificaron aquellos que requerirían mayor discusión/adaptación antes de ser aplicados en la Región.

En el segundo día de actividades, los participantes se enfocaron en la discusión de los principios de buenas prácticas identificados como prioritarios el día anterior, analizando experiencias de implementación de estos principios, retos y oportunidades para impulsarlos, requisitos y condiciones que deberían existir en el sistema de salud para que pudiesen implementarse, y recomendaciones generales para impulsar las buenas prácticas en la región.

En ambos días, las sesiones de trabajo en grupos fueron seguidas de sesiones plenarias para la presentación y discusión de los resultados obtenidos.

Una vez finalizado el Foro, la secretaría científica realizó un reporte final resumiendo las actividades, resultados y conclusiones. Tras recibir los comentarios y sugerencias de los participantes, se elaboró el reporte final.

## RESULTADOS

El Foro contó con un total de 42 participantes: 11 representantes de agencias de ETESA; 7 representantes de financiadores públicos, de la seguridad social y privados; un representante de la Organización Panamericana de la Salud; 7 académicos e integrantes de la secretaría científica; y 16 representantes de la industria (de fármacos, equipos médicos y test diagnósticos). En total estuvieron representados 10 países de la región. En el cuadro 1 se resumen las características principales de los sistemas de salud de estos países.

### Resultados de las presentaciones introductorias de los participantes

Los representantes de los países participantes expusieron las principales motivaciones que impulsaron el desarrollo de ETESA en sus países, y los principales desafíos que actualmente enfrentan. La heterogeneidad en los métodos utilizados en la toma de decisiones y las inequidades y heterogeneidad en el acceso fueron los aspectos más mencionados en lo referente a la motivación para impulsar el desarrollo de ETESA y centralizar la toma de decisiones. Otra motivación mencionada fue la necesidad de incrementar la legitimidad del proceso de toma de decisiones sobre asignación de recursos sanitarios. Entre los desafíos identificados con más frecuencia se encontraban la falta de recursos suficientes o de capacidad técnica, así como la falta de divulgación y conocimiento del tema por parte de la sociedad en general.

Desde la perspectiva de la industria de medicamentos, se mencionó la dificultad que implica el hecho de que exista tanta diversidad en los procesos de ETESA, en las reglas de decisión, en los umbrales de costo efectividad y en los requisitos de información por los países. Se consideró que los principales desafíos son incorporar la voz del paciente en el proceso de toma de decisiones y abogar por una mayor transparencia en los procesos de decisión.

Desde la industria de dispositivos, se resaltó el hecho de que los dispositivos médicos muchas veces no pasan por un proceso de ETESA y que es habitual que gran parte de la evidencia surja después del uso, a diferencia de lo que sucede con los medicamentos. Se plantearon como desafíos para un rol más protagónico de la ETESA, la falta de comunicación entre aquellos que evalúan y los responsables de la decisión de compra, y una estrategia de compra más basada en el precio y no en el valor de la tecnología. Se consideró como una oportunidad el surgimiento de iniciativas de ETESA a nivel hospitalario.

### Resultados de las discusiones grupales y sesiones plenarias sobre los principios de buenas prácticas

Mediante la estrategia de búsqueda descripta se identificaron y seleccionaron ocho publicaciones (cuadro 2) en las cuales se describían más de 50 principios de buenas prácticas para el desarrollo y uso de ETESA, los cuales sirvieron de base para el trabajo grupal y discusiones plenarias posteriores.

Aun reconociendo la heterogeneidad de desarrollo de la ETESA entre países de la región y las diferencias contextuales de sus sistemas sanitarios, cinco principios fueron identificados por los grupos como prioritarios y que tendrían mayor potencial para ser aplicados y/o profundizados en la región en el corto plazo (cuadro 3).

Se describen a continuación los principales puntos discutidos respecto a estos cinco principios:

### 1) Transparencia en procesos de realización ETESA y de comunicación de sus resultados

Todos los grupos coincidieron en que el proceso de ETESA debe ser transparente y libre de sesgo. En general hubo acuerdo en considerar que implementar un proceso de consulta pública, a través del cual los documentos de evaluación y/o decisión se publiquen en sus diferentes etapas de desarrollo, para recibir retroalimentación del público en general o de otros actores como profesionales de la salud o productores, sería una medida básica y relativamente accesible para las agencias, lo que le permitiría mejorar este aspecto. Brasil y Colombia fueron mencionados como ejemplos de implementación exitosa de mecanismos para incrementar la transparencia.

### 2) Involucramiento de los actores relevantes en el proceso de ETESA

Algunos de los grupos consideraron que este principio debía implementarse sin demoras ya que resulta imprescindible para darle legitimidad al proceso de ETESA y a la toma de decisiones, y en consecuencia reduciría el riesgo de conflictos y/o apelaciones judiciales. Se mencionó también la importancia de que este involucramiento (incluyendo a pacientes, usuarios, profesionales de la salud, decisores y otras partes interesadas como la industria) suceda desde el inicio del proceso de ETESA. Sin embargo, otros participantes expresaron que no resultaba tan prioritario, ya que la legitimidad se puede alcanzar por otros medios, por ejemplo, a través del involucramiento de sociedades científicas. Por otro lado, consideraron también que el involucramiento de partes interesadas puede ser complejo, requiere metodologías adecuadas y puede exponer al proceso de ETESA a influencias no deseadas (por ejemplo, a través de grupos de pacientes financiados por agentes interesados). Puede también representar una carga excesiva de trabajo y demorar los procesos de ETESA impidiendo dar respuestas a tiempo a los decisores. Nuevamente, Brasil y Colombia fueron mencionados como ejemplos de implementación exitosa de mecanismos para involucrar a distintos actores.

**CUADRO 1.** Resumen de las características de los países participantes

País	Población aproximada	Producto bruto interno (PBI), 2014. Millones de USD.	Gasto público en Salud (% del PBI)	Instituciones que desarrollan ETESA
Argentina	42 millones	526 320	2,7	Ministerio de Salud, Instituciones asociadas al Ministerio de Salud, gobiernos regionales, unidades hospitalarias, Universidades Universidad u organizaciones independientes con o sin fines de lucro.
Brasil	205 millones	2 455 993	3,8	Ministerio de Salud (CONITEC), gobiernos regionales, unidades hospitalarias, Universidades u organizaciones independientes con o sin fines de lucro.
Chile	18 millones	258 733	3,9	Departamento de Evaluaciones de Tecnologías Sanitarias del Ministerio de Salud, Universidades u organizaciones y organizaciones sin fines de lucro.
Colombia	49 millones	378 416	5,4	Institución asociada al Ministerio de Salud (IETS)
Costa Rica	5 millones	50 168	6,8	Institución asociada al Gobierno nacional o al Ministerio de Salud (CajaCostarricense del Seguro Social) y otras organizaciones independientes (Cochrane)
Ecuador	16,5 millones	102 292	4,5	Institución asociada al Gobierno nacional o al Ministerio de Salud
El Salvador	6,1 millones	25 054	4,5	Ministerio de Salud (Dirección de Tecnologías Sanitarias)
México	122 millones	1 298 176	6,1	Instituciones asociadas al Gobierno nacional o al Ministerio de Salud (CENETECSalud,IMSS, ISSSTE)
Perú	31 millones	201 021	3,3	Ministerio de Salud (Dirección General de Medicamentos Insumos y Drogas–DIGEMID, Unidad de Análisis y Generación de Evidencias en Salud Pública del Instituto Nacional de Salud–UNAGESP, Seguro Integral de Salud - GREP), Instituciones asociadas al Gobierno nacional o al Ministerio de Salud (Seguro Social de Salud, Instituto de Evaluación en Tecnologías en Salud e Investigación – IETSI) y Universidades. Ministerio de Salud e
Uruguay	3,3 millones	57 236	3,3	Instituciones asociadas al Gobierno nacional o al Ministerio de Salud (Fondo Nacional de Recursos)

***Fuente:*** Datos Banco Mundial, 2014, Disponible en: http://data.worldbank.org y encuesta completada por asistentes.

**CUADRO 2.** Documentos seleccionados que incluyen principios considerados de buenas prácticas para guiar el desarrollo y uso de las ETESA

Título		Institución/Grupo	Año
1.	Responsabilidad por razonabilidad/Justicia, salud y atención a la salud (Accountability for reasonableness)	Daniels y cols.	2000/2001
2.	ETESA para dispositivos médicos en Europa: Lo que debe tenerse en cuenta	Siebert y cols. para Eucomed	2002
3.	Principios de la Asociación Europea de la Industria Farmacéutica Innovadora (EFPIA).	EFPIA	2005
4.	Elementos esenciales de una Iniciativa de Evaluación de Tecnologías y Desenlaces	Emanuel y cols.	2007
5.	Principios clave para la mejora en la conducción de evaluaciones de tecnología sanitaria para la asignación de recursos del Grupo Internacional para el Avance de las ETESA (“Key Principles”)	The International Group for HTA Advancement	2008
6.	¿Cómo puede mejorarse el impacto de las evaluaciones de tecnología sanitaria?	Organización Mundial de la Salud (OMS) en representación del Observatorio Europeo de Sistemas y Políticas de Salud.	2008
7.	Principios de Buena práctica para las evaluaciones de efectividad relativa	High Level Pharmaceutical Forum	2008
8.	Tomando decisiones justas en el camino hacia la cobertura universal de la salud	Grupo consultor de la OMS sobre Equidad y Cobertura Universal de la Salud	2014

**ETESA:** Evaluación de Tecnologías Sanitarias.

**CUADRO 3.** Principios identificados como prioritarios para ser aplicados en América Latina y el Caribe

Principio
Transparencia en procesos de realización ETESA y de comunicación de sus resultados
Involucramiento de los actores relevantes en el proceso de ETESA
Existencia de mecanismos de apelación de las decisiones
Existencia de mecanismos claros para el establecimiento de prioridades en ETESA
Existencia de un vínculo claro entre la evaluación y la toma de decisión

### 3) Existencia de mecanismos de apelación

Son los mecanismos que permiten que las diferentes partes interesadas puedan apelar los resultados de la decisión que se ha tomado como parte del proceso de ETESA (por ejemplo, sobre la cobertura o inclusión de una determinada intervención en un paquete de cobertura). Algunos participantes coincidieron en que no es un aspecto complejo y que es necesario implementarlo sin demoras, ya que en muchos países de hecho debería formar parte de todo acto administrativo; otros participantes en cambio opinaron que es algo complejo y que puede entorpecer el proceso por dejarlo expuesto a apelaciones sin fundamento, y debilitar de esta forma las recomendaciones del Ministerio o agencia.

### 4) Existencia de mecanismos claros para el establecimiento de prioridades de los temas a evaluar por la ETESA

En este punto también hubo diferentes visiones. Se mencionó la opción de permitir que la industria solicite evaluaciones como en el caso de Brasil o México. Algunos participantes consideraron que, debido a los limitados recursos para realizar evaluaciones, esto no es una opción en su país y deben limitar los pedidos de evaluación a los pedidos internos de los organismos públicos. Algunos mencionaron la importancia de tener prioridades claramente definidas que les permitan manejar su agenda de evaluaciones según lo que es importante para el país y de esta forma evitar que la agenda de la agencia termine siendo manejada por las necesidades de la industria con sus pedidos de evaluación, o por contingencias dictadas por urgencias políticas. Por el contrario, otros participantes recomendaron no tener un proceso explícito de fijar prioridades. Consideran que el mismo rápidamente se tornaría incumplible dado que es inevitable que la agencia deba adaptar su agenda a diferentes grupos de presión, por ejemplo, para actualizar el plan de beneficios, o cuestiones que surgen de los tiempos políticos.

### 5) Existencia de un vínculo claro entre la evaluación y la toma de decisión

Este fue el principio en el cual se detectó mayor heterogeneidad entre los países. La vinculación entre el informe de ETESA y la toma de decisión no es muchas veces un proceso claro dentro del sistema de salud. La existencia de un marco legal apropiado que defina el rol de la agencia de ETESA fue mencionado como un factor decisivo para determinar este grado de vinculación, aunque no todos los participantes coincidieron en que aumentar esta vinculación fuese siempre deseable y/o posible en sus sistemas de salud. Un factor limitante puede darse en el caso de una agencia de ETESA que centraliza las evaluaciones en el contexto de un sistema de salud fragmentado donde podrían tomarse diferentes decisiones en diferentes niveles. Se mencionaron los casos de Brasil y México, como ejemplos en los cuales el rol de los informes de ETESA en el proceso de toma de decisiones está más claramente establecido, al existir un marco normativo específico.

Durante la discusión en los grupos y la discusión plenaria sobre estos principios surgieron como temáticas recurrentes la falta de recursos (principalmente recursos técnicos, de tiempo y presupuestarios) para la correcta aplicación de estos principios. Esto fue mencionado especialmente para los principios de transparencia del proceso y el involucramiento de actores, a los que se identificó como los principios que requerirían la mayor inversión de recursos. Se mencionó considerar la opción de obtener financiamiento a través de recursos de la industria, como es el caso de algunos países de Europa donde los productores de tecnologías deben pagar a la agencia por las evaluaciones, aunque varios participantes expresaron su preocupación respecto a esta opción por la mayor dificultad que existe en la región para el manejo de los conflictos de interés. Las presiones que pueden ejercer las diferentes partes interesadas y la presencia de conflictos de interés fueron mencionadas también como los retos fundamentales para la aplicación de algunos de los principios priorizados, por ejemplo, el involucramiento de actores, a los cuales se suma también el incremento de las demandas de tiempo y recursos que la aplicación de estos principios puede representar. Se mencionó la alternativa de impulsar iniciativas regionales para coordinar recursos y acciones de varios países de forma de reducir la carga de trabajo que la implementación de algunos de los principios podría implicar.

En la discusión varios participantes coincidieron en mencionar que todos los principios tienen matices y diferentes enfoques posibles, así como diferentes alcances o grados de profundidad, y por lo tanto es válido intentar avanzar de forma gradual, sin sentirse obligados a aplicar un “estándar NICE” desde el inicio.

## DISCUSIÓN

En este manuscrito presentamos los resultados del primer foro de estas características que se realiza en la región, en el cual evaluadores, financiadores y productores de tecnologías compartieron un espacio común para discutir temas de interés estratégico en ETESA.

La principal conclusión de este Foro, compartida por la mayoría de los participantes, es que a pesar de los grandes avances en la ETESA observados en la región en estos últimos años, existen todavía amplias oportunidades de mejora, sobre todo en aspectos relacionados con la aplicación de la ETESA al proceso de toma de decisiones.

Hubo coincidencia en que los principios de buenas prácticas postulados a nivel internacional (6–14) son en general válidos y potencialmente aplicables en LAC; y pueden resultar de utilidad para guiar los procesos de establecimiento, ampliación o mejora de los procesos de ETESA en la región. Sin embargo, la mayor parte de ellos requiere una adecuación al contexto local, y la decisión de cuáles de ellos y en qué medida deben ser implementados depende del estado del desarrollo de la ETESA en cada país, los recursos disponibles y las características del sistema de salud y el proceso de toma de decisiones. A su vez, existen experiencias importantes en la región (5), en las que varios países ya han comenzado a profundizar la aplicación de muchos de estos principios en sus procesos de ETESA, y estas experiencias pueden servir de guía al resto de los países.

Si bien en este Foro se hizo un esfuerzo por incluir una amplia gama de participantes, no todos los países de la región estuvieron representados, y los países y los sistemas de la región presentan una gran heterogeneidad. Estos factores representan una limitación a la generalización de nuestros hallazgos. Sin embargo, la discusión que se generó en este Foro sobre como determinar qué principios son más prioritarios, y de qué forma deben ser adaptados al contexto local, es coincidente con una preocupación que también existe a nivel global. Un documento reciente de la OMS (24) remarca la confusión y la dificultad para determinar cuál es la mejor forma de implementar la ETESA en los sistemas de salud y su uso en la toma de decisiones. La forma en que las agencias de ETESA aplican estos principios de buena práctica también varía a lo largo del mundo. Neumann y cols. encontraron una importante variabilidad en el grado en que una serie de principios eran aceptados e implementados en 14 organizaciones de ETESA, sobre todo en las áreas de transparencia en la articulación entre los resultados de la ETESA y la toma de decisiones. Un estudio realizado en el 2010 entre decisores de salud de LAC (25) ya había encontrado que los principios postulados a nivel internacional eran también considerados como válidos y relevantes en la región y, coincidentemente con los resultados de este Foro, halló también que las mayores brechas se encontraban en el grupo de principios relacionados con la aplicación de la ETE- SA en la toma de decisiones.

Los principales desafíos identificados por los países de este Foro para las iniciativas de ETESA fueron las dificultades en la inclusión de los distintos actores en el proceso, la falta recursos para el desarrollo y sostén de la capacidad técnica y la inadecuada diseminación y articulación de los resultados de la ETESA con la toma de decisiones. El principal reto identificado fue acerca de cómo ser eficientes en este proceso, y encontrar un equilibrio adecuado entre las mejoras planteadas en los procesos de ETESA y los recursos disponibles en los países (de personal, presupuesto y tiempo) sobre todo para prevenir que las mejoras a introducir en los procesos atenten contra los tiempos de producción de informes y la adecuación a las necesidades de los decisores. Estas advertencias respecto a los dilemas y potenciales problemas con los que puede enfrentarse una agencia a la hora de intentar aplicar o profundizar principios de buenas prácticas postulados a nivel global, coinciden con las críticas y advertencias que cada postulación de principios ha generado en la comunidad global de ETESA (15–20).

Los participantes coincidieron en que los próximos pasos a seguir deberían ir dirigidos a impulsar los criterios priorizados. Esto permitirá aumentar la legitimidad de las difíciles decisiones que los sistemas de salud deben tomar en el camino a la cobertura universal en salud. La principal recomendación fue encontrar los procesos y/o metodologías de ETESA adecuados, adaptados al contexto de cada país, que permitan avanzar gradualmente en mejorar el vínculo existente entre la ETESA y la toma de decisiones; sin pretender imponer a corto plazo estándares excesivamente elevados tomados de ejemplos a nivel internacional sin la adecuada adaptación al contexto local.

### Agradecimientos.

A los participantes del evento: Anmol Mullins (Alcon), Mariana Naranjo Muedano (AMGEN), Sean Nagle (Novartis), Adrian Griffin (Johnson & Johnson), Alarico Rodríguez (Fondo Nacional de Recursos - Uruguay), Albin Chaves Matamoros (Caja costarricense del Seguro Social - Costa Rica), Alexandre Lemgruber (OPS), Alicia Granados (Genzyme), Ana Eduviges Sancho Jiménez (Ministerio de Salud - Costa Rica), Ana Pérez Galán (Ministerio de Salud - Uruguay), Antoine Wolniewicz (Genzyme), Bernardo Tinajero (Novartis), Andrew Bruce (AMGEN), Carlo Crisostomo (Johnson & Johnson), Chris Henshall (HTAi), Clarice Alegre Petramale. (CONITEC -Brasil), Diego Guarín (Merck), Elsa Koutsavakis. (Pfizer), Giovanni Francisco Guevara Vásquez: (Ministerio de Salud - El Salvador), Guillermo Sánchez Vanegas (IETS-Colombia), Homero Monsanto (Merck), Jens Grueger (Roche), Jocelyn Ramírez (Eli Lilly), Joice Valentim (Roche), José Luis Estrada Aguilar (IMSS - México), Joseph Cook (Pfizer), Lizbeth Acuña (Cuenta de Alto Costo - Colombia), Luis Gamero (Mnisterio de Salud - Perú), Manny Papadimitropoulos (Eli Lilly), María Luciana Aramijos Acurio (Ministerio de Salud - ECUA- DOR), Mariana Pineda (CENETEC - México), Marianela Castillo Riquelme (Ministerio de Salud -Chile), Mauricio Duarte Ruano (Caja costarricense del Seguro Social - Costa Rica), Rafael Zamora (PAMI -Argentina), Rubén Agustín Nieto (UCEETS -Argentina), Sean Tunis (HTAi), Sir Andrew Dillon (NICE-Reino Unido), Stefan Weber (Novartis International - Alcon), Virginia Baffigo Torre de Pinillos (ESSALUD - Perú),

### Financiación.

La organización y asistencia al Foro fue financiada por la Sociedad Científica Internacional de Evaluación de Tecnología Sanitaria (HTAi). La preparación del manuscrito estuvo a cargo del Instituto de Efectividad Clínica y Sanitaria (IECS) con financiamiento de Health Technology Assessment International (HTAi).

### Declaración.

Las opiniones expresadas en este manuscrito son responsabilidad del autor y no reflejan necesariamente los criterios ni la política de la *RPSP/PA-* *JPH* y/o de la OPS.
